# Islet-like organoids derived from human pluripotent stem cells efficiently function in the glucose responsiveness *in vitro* and *in vivo*

**DOI:** 10.1038/srep35145

**Published:** 2016-10-12

**Authors:** Youngjin Kim, Hyeongseok Kim, Ung Hyun Ko, Youjin Oh, Ajin Lim, Jong-Woo Sohn, Jennifer H. Shin, Hail Kim, Yong-Mahn Han

**Affiliations:** 1 Department of Biological Sciences, KAIST, Daejeon 34141, Republic of Korea; 2 Graduate School of Medical Science and Engineering, KAIST, Daejeon 34141, Republic of Korea; 3 Department of Mechanical Engineering, KAIST, Daejeon, 34141, Republic of Korea

## Abstract

Insulin secretion is elaborately modulated in pancreatic ß cells within islets of three-dimensional (3D) structures. Using human pluripotent stem cells (hPSCs) to develop islet-like structures with insulin-producing ß cells for the treatment of diabetes is challenging. Here, we report that pancreatic islet-like clusters derived from hESCs are functionally capable of glucose-responsive insulin secretion as well as therapeutic effects. Pancreatic hormone-expressing endocrine cells (ECs) were differentiated from hESCs using a step-wise protocol. The hESC-derived ECs expressed pancreatic endocrine hormones, such as insulin, somatostatin, and pancreatic polypeptide. Notably, dissociated ECs autonomously aggregated to form islet-like, 3D structures of consistent sizes (100–150 μm in diameter). These EC clusters (ECCs) enhanced insulin secretion in response to glucose stimulus and potassium channel inhibition *in vitro*. Furthermore, ß cell-deficient mice transplanted with ECCs survived for more than 40 d while retaining a normal blood glucose level to some extent. The expression of pancreatic endocrine hormones was observed in tissues transplanted with ECCs. In addition, ECCs could be generated from human induced pluripotent stem cells. These results suggest that hPSC-derived, islet-like clusters may be alternative therapeutic cell sources for treating diabetes.

Diabetes mellitus (DM) is a metabolic disease characterized by hyperglycemia. DM can cause a variety of complications, including cardiovascular disease, stroke, blindness, and renal failure^1^,^2^. In general, DM is categorized as either type 1 or 2, according to its heterogeneous etiology. Type 1 DM is an autoimmune disease with ß cell destruction, and type 2 DM is caused by peripheral insulin resistance with insufficient insulin production in ß cells^3^,^4^. Because β cell insufficiency is a common feature of DM, the replenishment of functional ß cell mass is a promising therapy for the treatment of DM^1^,^5^,^6^. However, transplantable ß cell sources for cell replacement therapy are extremely limited because of a lack of donor organs^7^.

Alternative approaches have been attempted, including the *in vivo* regeneration of ß cells in the pancreas and the *in vitro* differentiation of human pluripotent stem cells (hPSCs) into insulin-producing ß cells. In the pancreas, stem cell-like cells, exocrine acinar cells, and pancreatic ductal cells are considered ß cell surrogates in either the pancreatectomy or ductal ligation model^8^,^9^,^10^. Recently, it has been reported that antral stomach cells can be converted into insulin-positive cells by the ectopic expression of ß cell reprogramming factors^11^. Nonetheless, as the ß cell-like cells obtained from those approaches are derived in non-physiological conditions, such as metabolic stress and injury response, or via cellular reprogramming, the generation of bona fide ß cells remains limited^10^. As alternatives, many approaches for differentiating hPSCs into insulin-producing cells have been attempted *in vitro*^12^,^13^,^14^,^15^,^16^. Although improved differentiation protocols to generate functionally mature β cells from hPSCs have been reported recently^17^,^18^,^19^, hPSC-derived β cells show polyhormonal expression, limited expression of mature β cell markers, and the lack of glucose-stimulated insulin secretion. Nonetheless, there are many restrictions for using transplantable hPSC-derived ß cells in therapeutic applications, including inefficient differentiation and a lack of functionality 6,20,21,22,23.

The islets of Langerhans, a pancreatic endocrine tissue, are composed of insulin-producing ß cells, glucagon-producing α cells, somatostatin-producing δ cells, pancreatic peptide-producing PP cells, and ghrelin-producing ε cells^24^,^25^,^26^,^27^,^28^. Distinct endocrine cells are organized into clusters to construct the islets of Langerhans, and individual endocrine cells functionally mature in the islet structure during the early post-neonatal period in mice^29^,^30^. Thereafter, endocrine cells in the pancreatic islets play roles in the regulation of blood glucose levels. Moreover, reciprocal interactions among endocrine cells in the islets are critical for the regulation of insulin secretion in response to glucose. The interplay between ß cells through gap junctions synchronizes heterogeneous glucose responsiveness; pancreatic endocrine hormones secreted from δ and α cells also regulate insulin secretion from ß cells^31^,^32^,^33^. Therefore, pancreatic islet structures would be effective means of physiologically regulating insulin secretion. Nonetheless, the development of hPSC-derived, islet-like structures has not yet been reported.

In this study, we demonstrate that pancreatic islet-like clusters generated from hESCs functionally secrete insulin. Endocrine cells (ECs) expressing pancreatic endocrine hormones were differentiated from hESCs using a step-wise protocol. Intriguingly, endocrine cell clusters (ECCs) formed spontaneously from dissociated ECs in one day. The size of the ECCs was approximately 50–150 μm in diameter, which is similar to that of the pancreatic islet. Transcriptional levels of ß cell-associated genes and glucose-stimulated insulin secretion were enhanced in ECCs compared to ECs. In addition, intracellular Ca^2+^ influx oscillated in ECCs during glucose stimulation. Moreover, diabetic mice transplanted with ECCs survived for approximately 2 months. Functional ECCs could also be derived from human induced pluripotent stem cells (hiPSCs). Thus, hPSC-derived ECCs exhibited ß cell-like functions both *in vitro* and *in vivo*. Our results indicate that hPSC-derived ECCs can effectively secrete insulin for the treatment of diabetes.

## Results

**Figure 1:**
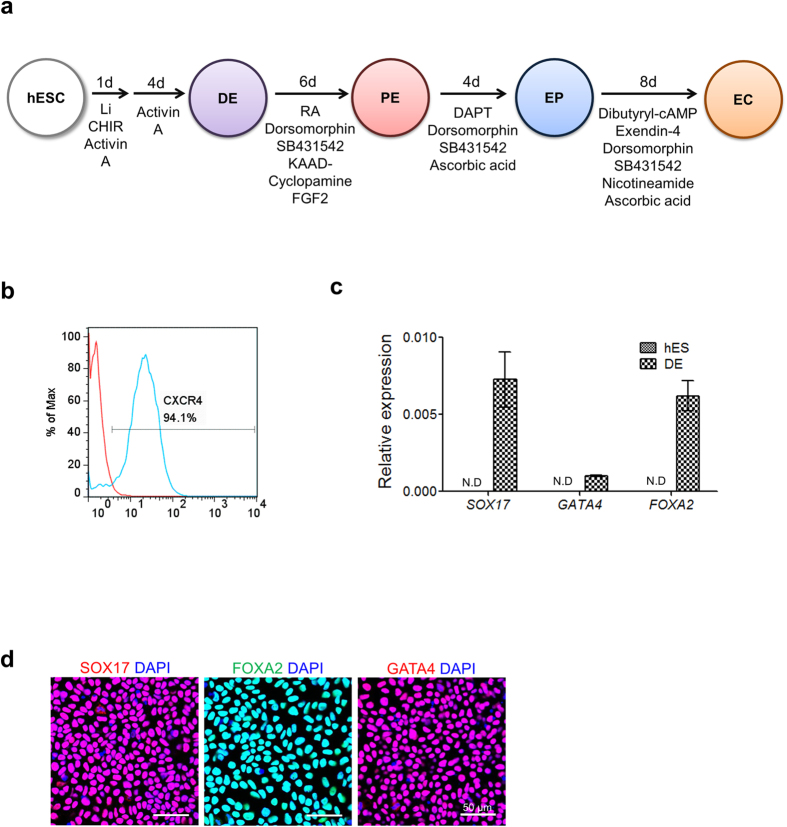
Overall scheme of hESC differentiation into definitive endoderm (DE) cells. (**a**) Overall protocols for the differentiation of human PSCs into pancreatic endocrine cells. DE, definitive endoderm; PE, pancreatic endoderm; EP, endocrine progenitor; EC, endocrine cell. (**b**) Flow cytometric analysis of CXCR4-positive cells differentiated from hESCs. CXCR4 was used as a DE marker. (**c**) Transcriptional expression of the DE marker genes *SOX17*, *GATA4* and *FOXA2.* Relative expression is represented as the mean ± SEM (n = 3). N.D., not detected. (**d**) Immunostaining of the representative DE markers SOX17, FOXA2 and GATA4 in hESC-derived DE cells. Nuclear DAPI staining is shown in blue. Scale bar, 50 μm.

Based on previous reports^12^,^13^,^14^,^15^,^16^,^34^,^35^, efficient protocols for differentiating hESCs into pancreatic endocrine cells have been re-established in this study (Fig. 1a). At the final step, ECCs were generated from hESC-derived ECs, and their functionalities were examined *in vitro* and *in vivo*.

### Differentiation of hESCs into pancreatic endocrine cells

To induce hESCs into definitive endoderm (DE) cells, hESCs were treated with a combination of activin A with CHIR99021 and LiCl for 1 day and were then treated with activin A alone for 4 days. As indicated in Fig. 1b, flow cytometric analysis showed a high proportion of CXCR4-positive cells (approximately 94%), representing the successful induction of hESCs to DE. DE markers, such as SOX17, FOXA2, and GATA4, were highly expressed in the hESC-derived DE cells at the mRNA (Fig. 1c) and protein levels (Fig. 1d). Thus, an optimal protocol for DE induction of hESCs was established in this study.

**Figure 2:**
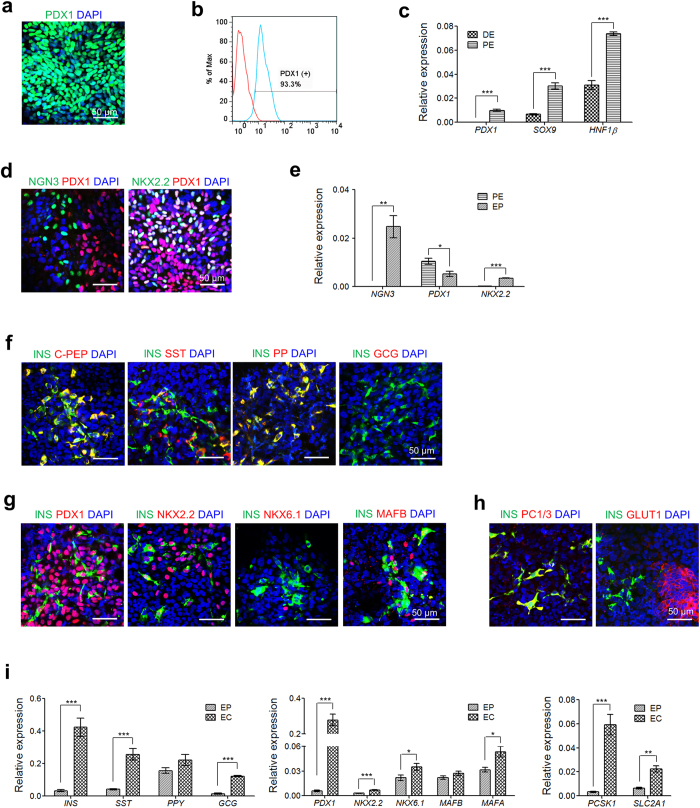
Differentiation of hESC-derived DE cells into hormone-expressing endocrine cells (ECs). (**a**) Immunostaining of the representative PE marker PDX1 in hESC-derived PE cells. Nuclear DAPI staining is shown in blue. Scale bar, 50 μm. (**b**) Flow cytometric analysis of PDX1-positive cells differentiated from hESCs. (**c**) Transcriptional expression of the PE marker genes *PDX1, SOX9* and *HNF1*ß. Relative expression is represented as the mean ± SEM (n = 3 or 4); *p < 0.05, **p < 0.01, ***p < 0.001. (**d**) Immunostaining for NGN3/PDX1 and NKX2.2/PDX1 in hESC-derived EP cells. Scale bar, 50 μm. (**e**) Transcriptional expression of *NGN3, PDX1,* and *NKX2.2*. Relative expression is represented as the mean ± SEM (n = 3 or 4); *p < 0.05, **p < 0.01, ***p < 0.001. (**f**) Expression of pancreatic endocrine hormones in hESC-derived ECs. INS, insulin; C-PEP, c-peptide; SST, somatostatin; PP, pancreatic polypeptide; GCG, glucagon. (**g**) Expression of ß cell-associated transcriptional factors in hESC-derived ECs. (**h**) Expression of ß cell function-related proteins in hESC-derived ECs. PC1/3, proprotein convertase 1/3; GLUT1, glucose transporter 1. Scale bar, 50 μm. (**i**) Transcriptional expression of pancreatic endocrine hormone genes (*INS*, *SST*, *PPY,* and *GCG)*, ß cell-associated transcriptional factor genes (*PDX1, NXX2.2*, *NKX6.1*, *MAFB*, and *MAFA*), and ß cell function-related genes (*PCSK1,* and *SLC2A1*). Relative expression is represented as the mean ± SEM (n = 3 or 4); *p < 0.05, **p < 0.01, ***p < 0.001.

Next, pancreatic endoderm (PE) specification was induced in DE cells by a combined treatment of retinoic acid (RA), dorsomorphin, SB432942, bFGF (Basic fibroblast growth factor), and KAAD-cyclopamine. hESC-derived PE cells robustly expressed a PE marker, PDX1 (Fig. 2a). Flow cytometric analysis consistently revealed a high proportion of PDX1-expressing cells (Fig. 2b), indicating successful PE specification after the DE stage. The transcripts of PE-related genes, such as *PDX1*, *SOX9,* and *HNF1*ß, were significantly enriched in PE cells compared with DE cells (Fig. 2c).

Hormone-producing endocrine cells (ECs) develop from PE cells through the endocrine progenitor (EP) stage, during which neurogenin3 (*NGN3*) is transiently expressed^30^. EP cells were obtained on day 4 of PE cell differentiation into ECs. hESC-derived EP cells expressed a representative EP marker, NGN3, and co-expressed NKX2.2 with PDX1 in the nucleus (Fig. 2d). The transcriptional expression levels of *NGN3* and *NKX2.2* were significantly enhanced, whereas *PDX1* expression was reduced at the EP stage compared with the levels at the PE stage (Fig. 2e).

Finally, hormone-expressing ECs were developed from EP cells. hESC-derived ECs appeared to be populated in boundaries and strongly expressed PDX1 in the nucleus (Supplementary Figure 1a). As shown in Fig. 2f and Supplementary Figure 1b, hESC-derived ECs expressed pancreatic endocrine hormones, including insulin (INS), somatostatin (SST), and pancreatic peptide (PP). Glucagon (GCG) was not detected in hESC-derived ECs. C-peptide (C-PEP) was clearly co-expressed with insulin in hESC-derived ECs, which indicates *de novo* insulin synthesis. Pancreatic ß cell-associated transcriptional factors (i.e., PDX1, NKX2.2, NKX6.1, and MAFB) were also expressed in hESC-derived ECs (Fig. 2g). Low expression of a mature ß cell marker, NKX6.1, and co-expression of MAFB with insulin were detected in hESC-derived ECs. These results suggest that the hESC-derived ECs were immature. Furthermore, the hESC-derived ECs expressed ß cell function-related proteins, such as proprotein convertase 1 (PC1/3) and glucose transporter 1 (GLUT1) (Fig. 2h). Similarly, the transcriptional activity of pancreatic endocrine hormone genes, ß cell-associated transcription factors, and ß cell function-related genes was enhanced in hESC-derived ECs compared with that in EP cells (Fig. 2i). Collectively, hESCs could differentiate into ECs expressing endocrine hormones. Nonetheless, these ECs appeared to be immature, as indicated by the marker expression profile.

### Clustering of ECs into a pancreatic islet-like structure

**Figure 3:**
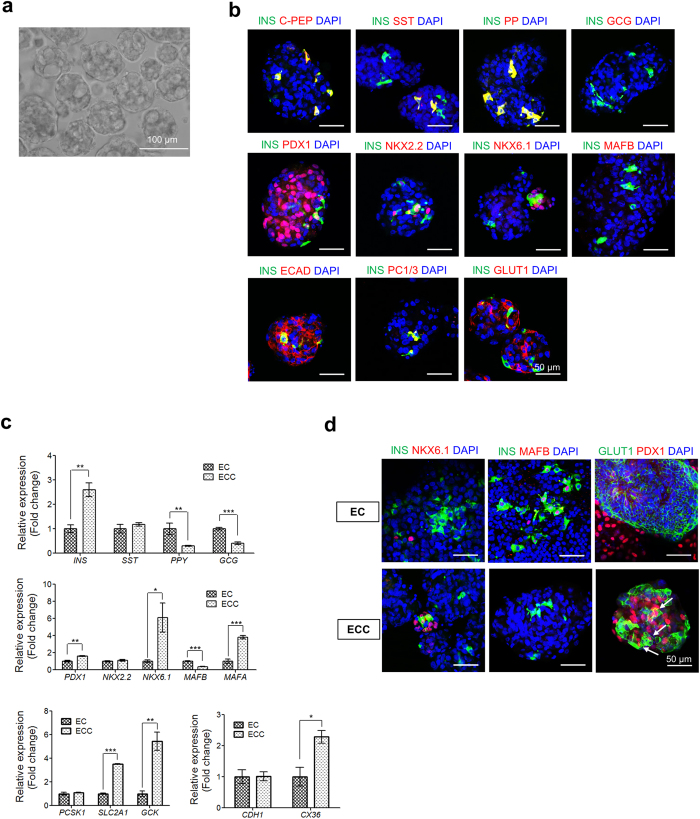
Efficient production of islet-like organoids derived from hESCs (hESC-derived ECCs). (**a**) Representative image of hESC-derived ECCs. Scale bar, 100 μm. (**b**) Expression of pancreatic endocrine hormones, ß cell-associated transcriptional factors, ß cell function-related proteins in hESC-derived ECCs. Scale bar, 50 μm. (**c**) Comparison of transcriptional levels of endocrine hormone genes (*INS*, *SST*, *PPY* and GCG), ß cell-associated transcriptional factor genes (*PDX1, NXX2.2*, *NKX6.1*, *MAFB*, and *MAFA*), ß cell function-related genes (*PCSK1, SLC2A1,* and *GCK*), and ß cell gap junction-related genes (*CDH1*, and *CX36*) between hESC-derived ECs and ECCs. (**d**) Maturation of hESC-derived, ß cell-like cells in hESC-derived ECCs. Co-expression of INS/NKX6.1 and GLUT1/PDX1 was only detected in hESC-derived ECCs, not in hESC-derived ECs. Scale bar, 50 μm.

Isolated mouse pancreatic ß cells have been shown to cluster *ex vivo* in culture^36^. To test whether hESC-derived ECs are also capable of clustering, 2D-cultured ECs were dissociated, placed on non-coated plates, and then incubated under static conditions. Surprisingly, pancreatic islet-like clusters formed from the dissociated ECs within 1 d after seeding (Fig. 3a). The size of each EC cluster (ECC) ranged from 50–150 μm. The optimal cell density for forming clusters with a definitive morphology was 5 × 10^4^ cells/well in a 96-well plate. Higher cell densities resulted in the formation of large and irregular clusters with debris; these cells seemed to become rapidly senescent (Supplementary Figure 2). The process of dissociated ECs clustering is shown in a movie (Supplementary Movie 1). As shown in Fig. 3b, hESC-derived ECCs expressed endocrine hormones (insulin, somatostatin, and pancreatic peptide) as well as ß cell transcription factors (PDX1, NKX2.2, and NKX6.1). Insulin-expressing cells clearly co-expressed c-peptide, whereas glucagon was not expressed in the ECCs (Fig. 3b, upper and Supplementary Figure 3a). Intriguingly, cells co-expressing insulin/NKX6.1 were observed in the ECCs, whereas MAFB was not detected in the insulin-expressing cells (Fig. 3b, middle). These results demonstrate that hESC-derived ECCs have ß cell-like properties. Insulin-expressing cells expressed E-cadherin (ECAD) in the membrane, and glucose transporter 1 expression was clearly observed in cell membranes within the ECCs (Fig. 3b, lower). Thus, we found that hESC-derived ECs could form pancreatic islet-like structures, or islet-like organoids, *in vitro* as a result of their own characteristics.

Next, the transcriptional activities of pancreatic ß cell-associated genes were investigated in hESC-derived ECCs (Fig. 3c). The expression of *INS* significantly increased, whereas *GCG* and *PPY* were not transcriptionally enhanced in the ECCs compared with ECs. The expression of mature ß cell marker genes, such as *PDX1, NKX6.1*, and *MAFA*, was transcriptionally activated after cluster formation, but *MAFB* expression was significantly reduced in the ECCs. The expression levels of ß cell glucose sensor genes (*SLC2A1* and *GCK*), and gap-junction genes (*CDH1* and *CX36*) were significantly enhanced in the ECCs. Thus, these results suggested that the clustering of hESC-derived ECs into pancreatic islet-like structures improved the potential of pancreatic ß cell maturation. This possible increment of ß cell maturation was confirmed by immunochemistry analyses (Fig. 3d). Co-expression of insulin with NKK6.1 was observed in hESC-derived ECCs, but not in ECs. Moreover, an endocrine progenitor marker, MAFB, was expressed in hESC-derived ECs, but not in hESC-derived ECCs. It has been reported that the non-functionality of hESC-derived endocrine cells may be due to the paucity of GLUT1 expression in insulin-expressing cells^22^. As shown in Fig. 3d, the hESC-derived ECCs contained cells co-expressing GLUT1/PDX1 (white arrows), whereas GLUT1 and PDX1 were separately expressed in different ECs. Orthogonal confocal microscopy images obviously showed the co-expression of GLUT1/PDX (Supplementary Figure 3b). Therefore, hESC-derived ECCs exhibit more properties of mature ß cells than do ECs.

### *In vitro* pancreatic function of hESC-derived ECCs

**Figure 4:**
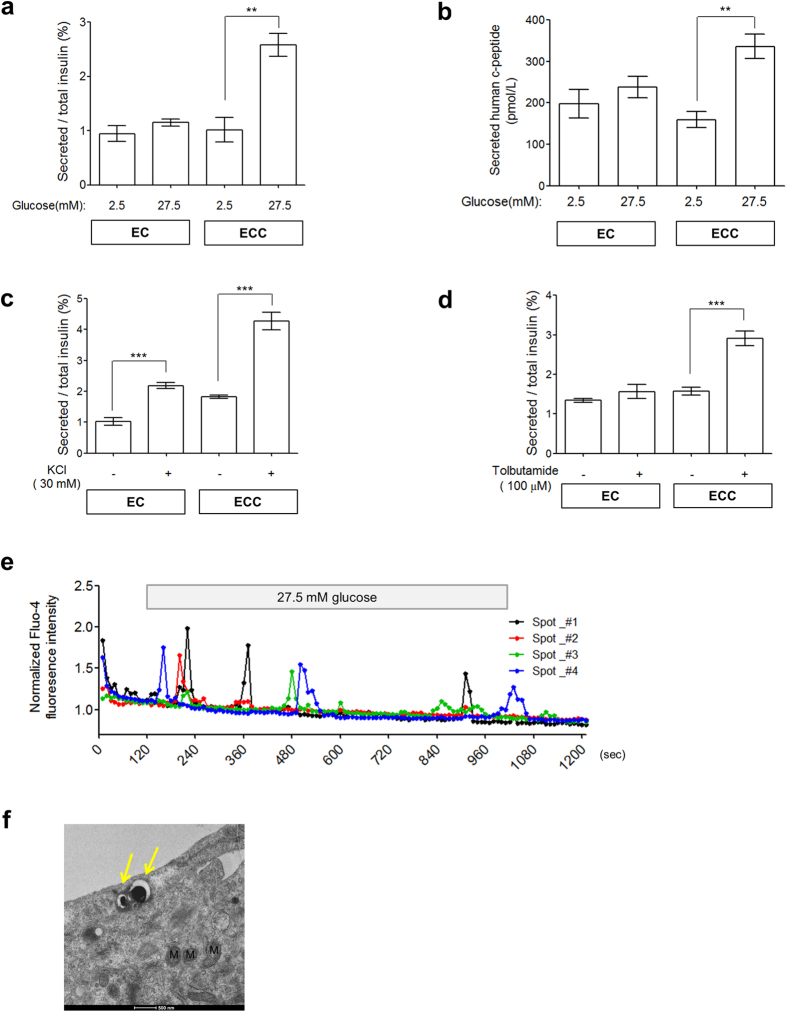
*In vitro* functionalities of islet-like organoids derived from hESCs. Responsiveness to 27.5 mM glucose stimulation of hESC-derived ECs and ECCs was analyzed by secretion levels of human insulin (**a**) and human c-peptide (**b**). Human insulin secreted in response to secretagogues, such as 30 mM KCl (**c**) and 100 μM tolbutamide (**d**) was measured. Secreted insulin and c-peptide levels are represented as the mean ± SEM (n = 3 or 4); *p < 0.05, **p < 0.01, ***p < 0.001. All secretion assays were performed after equilibration in 2.5 mM glucose. (**e**) Intracellular Ca^2+^ traces from hESC-derived ECCs. Detection of intracellular Ca^2+^ was measured using Fluo-4 AM. Responsive spots within ECCs were independently noted during the overall procedure. A representative spot is indicated by a yellow square in Supplementary Movie 2. (**f**) Transmission electron microscopy images of hESC-derived ECCs. Insulin-containing granules are indicated by yellow arrows. M, mitochondria. Scale bar, 500 nm.

Pancreatic ß cells in the islets acquire competence for glucose-responsive insulin secretion during the early postnatal period^29^,^30^. To analyze the functionality of hESC-derived ECCs, we first examined their ability to secrete insulin. As indicated in Fig. 4a, hESC-derived ECCs showed significantly increased insulin secretion with high glucose concentrations (1.01 ± 0.22% vs 2.6 ± 0.21%, P < 0.005, n = 4), whereas hESC-derived ECs did not (0.95 ± 0.15% vs 1.15 ± 0.067%, P = 0.2635, n = 4). Consistently, only ECCs exhibited significant secretion of human c-peptide after high glucose stimulation (159.6 ± 20.01 pmol/L vs 336.3 ± 29.21 pmol/L, P < 0.005, n = 3), whereas ECs did not show glucose-responsive c-peptide secretion (197.8 ± 34.75 pmol/L vs 237.7 ± 25.85 pmol/L, P = 0.4091, n = 3) (Fig. 4b). These results indicate that hESC-derived ECCs are capable of glucose-stimulated insulin secretion, which represents functional maturation. As shown in Fig. 4c, both hESC-derived ECs and ECCs significantly secreted insulin with low glucose stimulation in response to KCl treatments (1.02 ± 0.12% vs 2.18 ± 0.1% for ECs and 1.88 ± 0.09% vs 4.28 ± 0.28% for ECCs, p < 0.001, n = 4, respectively). Intriguingly, hESC-derived ECCs responded to K_ATP_ channel inhibition via a tolbutamide treatment (Fig. 4d); only ECCs significantly secreted insulin (1.57 ± 0.1% vs 2.90 ± 0.18%, P < 0.001, n = 4), whereas ECs did not (1.34 ± 0.048 vs 1.56 ± 0.18%, P = 0.2687, n = 4). These results suggest that insulin secretion from hESC-derived ECCs is regulated via K_ATP_ channels.

Glucose-induced insulin exocytosis involves an abrupt increase in [Ca^2+^]_*i*_ in pancreatic ß cells^31^,^37^. In this study, a small population of cells within the ECCs exhibited [Ca^2+^]_*i*_ oscillation of different intensities and times during high glucose stimulation (Fig. 4e). The first [Ca^2+^]_*i*_ influx occurred within 2 min after the glucose treatment. The real-time [Ca^2+^]_*i*_ influx is shown in Supplementary Movie 2. Electron microscopy (EM) revealed that the hESC-derived cells have insulin-containing vesicles (Fig. 4f, yellow arrows). This observation is consistent with those of typical spherical organelles containing an electron-dense core with a halo observed in mature ß cells, as previously reported^18^,^38^. Consequently, our results demonstrate that hESC-derived ECCs have the pancreatic ß cell-like function of glucose-responsive insulin secretion.

### Therapeutic effects of hESC-derived ECCs in a diabetic mouse model

**Figure 5:**
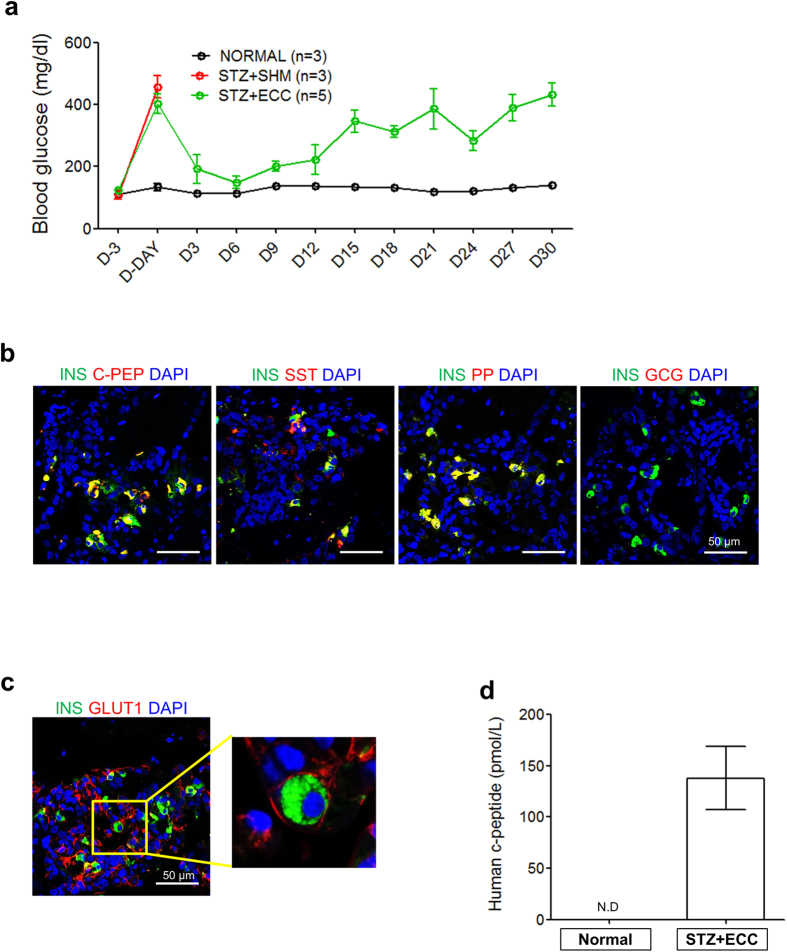
Therapeutic effects of hESC-derived ECCs in ß cell-deficient mice. (**a**) Regulation of blood glucose levels in STZ-treated mice after transplantation with hESC-derived ECCs. The ß cell-deficient mice were produced by treatment with STZ. Blood glucose levels were measured in mice every 3 d after 4 h of fasting. The data are represented as the mean ± SEM. (**b**) Expression of pancreatic endocrine hormones in tissues engrafted with hESC-derived ECCs. Tissues were obtained from mice that were euthanized 13 d after the transplantation of hESC-derived ECCs; these tissues were immunostained with antibodies against respective endocrine hormones. Nuclear DAPI staining is shown in blue. Scale bar, 50 μm. (**c**) Co-expression of INS/GLUT1 in engrafted hESC-derived ECCs. This result demonstrates the *in vivo* functionality of engrafted hESC-derived ECCs for blood glucose regulation in ß cell-deficient mice. Scale bars, 50 μm. (**d**) Secretion of human c-peptide in the serum of mice transplanted with hESC-derived ECCs. Serum was collected 12 d after the transplantation of hESC-derived ECCs. Measured c-peptide levels are represented as the mean ± SEM (n = 3). N.D., not detected.

Finally, we examined the *in vivo* functionality of hESC-derived ECCs. A number of ECCs (2.2~3.8 × 10^4^ ECCs/mouse) were transplanted into the epididymal fat pads of NOD/SCID mice. To induce diabetes, streptozotocin (STZ, 175 mg/kg) was injected intraperitoneally 3 d before transplantation. The functionality of the transplanted ECCs was first evaluated by the survival and blood glucose levels of the STZ-treated mice. The STZ treatment destroyed the pancreatic islets of the mice (Supplementary Figure 4a). For the survival of STZ-treated mice, long-acting insulin (0.15 U/kg body weight) was subcutaneously administered until transplantation. Sham-operated mice died due to hyperglycemia 3 d after the withdrawal of insulin treatment, whereas ECC-transplanted mice survived more than 40 d (Fig. 5a). The blood glucose level in ECC-transplanted mice rapidly decreased to within the normal ranges observed in the control mice within 3 d post-transplantation. When the ECC-transplanted mice were euthanized 12 d after transplantation, endocrine hormones, such as insulin, c-peptide, somatostatin, and pancreatic polypeptide, were observed in the ECC-transplanted tissues using immunochemistry (Fig. 5b). Furthermore, the insulin-expressing cells exhibited GLUT1–anchored membranes (Fig. 5c). Moreover, human c-peptide (137.9 ± 31.06 pmol/L, P < 0.005, n = 3) was detected in the blood of ECC-transplanted mice 12 d after transplantation (Fig. 5d). Unexpectedly, the ECC-transplanted mice showed uncontrolled hyperglycemia after 12 d post-operation, and the concentration of human c-peptide in the blood was reduced (20.19 ± 1.33 pmol/L, P < 0.005, n = 2) (Supplementary Figure 4d). The hyperglycemia could be attributed to dysfunction of the transplanted ECCs. At 13 d, PDX1-positive immunostaining was detected in the cytoplasm, not in the nuclei, of cells in autopsied ECC-transplanted tissue (Supplementary Figure 4b). Additionally, ECC-transplanted tissue autopsied at 49 d did not show insulin/PDX1 expression (Supplementary Figure 4c). These results demonstrate that transplanted hESC-derived ECCs have the pancreatic ß cell-like function of controlling the blood glucose level via glucose-sensing GLUT1 in diabetic mice. Pancreatic islet-like structures could also be produced from ECs developed from another hESC line, CHA15-hESCs (Supplementary Figure 5), and a hiPSC line (Supplementary Figure 6). Taken together, our findings suggest that hPSC-derived ECCs are capable of pancreatic ß cell-like functions both *in vitro* and *in vivo.*

## Discussion

Here, we report for the first time that functional pancreatic islet-like organoids can be generated from hPSCs. Notably, hPSC-derived ECCs efficiently secreted insulin in response to glucose *in vitro*, as do pancreatic ß cells. Additionally, insulin-deficient mice transplanted with hESC-derived ECCs survived for a long time (i.e., more than 40 d) and were capable of blood glucose level regulation. Insulin-producing cells have been generated from hPSCs *in vitro*^12^,^13^,^14^,^15^,^16^,^22^,^35^,^39^. However, hPSC-derived, insulin-producing cells exhibit poly-hormonal expression, insufficient glucose-responsiveness, heterogeneous populations, and fetal ß cell-like gene signatures^20^,^23^. Those insufficient functionalities might conceivably be due to an incomplete microenvironment around the hPSC-derived, insulin-producing cells, unlike that of ß cells within pancreatic islets. The functional maturation of ß cells is completed within pancreatic islets during the early post-neonatal period^29^,^30^. In this study, hPSC-derived, 3D structures were found to mimic pancreatic islets to some extent in terms of ß cell maturation, glucose responsiveness *in vitro*, and blood glucose level regulation in diabetic mice.

As previously reported^20^,^23^, insulin-expressing ECs differentiated from hESCs appeared to be immature in terms of endocrine marker expression patterns and glucose responsiveness. In this study, hESC-derived ECs did not co-express INS/NKX6.1 and INS/GLUT1, and they expressed an endocrine progenitor marker, MAFB (Fig. 2g,h). Furthermore, the secretion of insulin and c-peptide was not significantly increased in hESC-derived ECs under high glucose concentrations (Fig. 4a,b) or potassium channel inhibition via a tolbutamide treatment (Fig. 4d), suggesting weak glucose responsiveness. In this study, the limitations of pancreatic endocrine cells derived from hESCs for insulin secretion were overcome in the hESC-derived ECCs.

Insulin-expressing ß cells have been reported to have self-clustering dynamics even in *ex vivo* culture systems^36^. Based on this concept, we attempted to make 3D structures using hESC-derived pancreatic endocrine cells. Surprisingly, hESC-derived ECs could spontaneously form 3D structures under optimal conditions (Supplementary Figure 2 and Supplementary Movie 1); these structures were referred to as ECCs. These hESC-derived ECCs exhibited higher transcriptional expression of pancreatic ß cell-associated genes than that of hESC-derived ECs (Fig. 3c). Intriguingly, the size of the hESC-derived ECCs ranged from approximately 50–150 μm in diameter with relative consistency (Fig. 3a). Islet size, which is approximately 100 μm in diameter, is relatively comparable among a variety of species, such as human, pig, mouse, and rabbit^40^, although the mechanism of the size limitation remains unclear. More interestingly, hESC-derived ECCs expressed pancreatic cell type-specific endocrine hormones (Fig. 3b): insulin and c-peptide, which are specific to ß cells; somatostatin, specific to δ cells; and pancreatic polypeptide, specific to PP cells. The hESC-derived ECCs comprised several pancreatic endocrine cell types, excluding α cells, and thus represent islet-like organoids. These results demonstrate that hESC-derived ECCs are, to some extent, analogous to human pancreatic islets in terms of size and cell composition.

Despite their insufficient endocrine cell composition, the hESC-derived ECCs exhibited insulin secretion similar to the corresponding functionality of pancreatic islets. Glucose triggering insulin secretion from ß cells involves a series of pathways and events, such as glucose metabolism, K_ATP_-channel closure, depolarization, and Ca^2+^ influx^37^,^41^,^42^. Intriguingly, compared with hESC-derived ECs, hESC-derived ECCs efficiently responded to glucose stimuli by secreting insulin (Fig. 4a) and c-peptide (Fig. 4b). The absence of glucose responsiveness in the hESC-derived ECs might be responsible for the ß cell immaturity *in vitro*. In fact, the hESC-derived ECs showed lower transcriptional levels of GLUT1 (*SLC2A1*) and did not co-express GLUT1/PDX1 (Fig. 3c,d, respectively). Additionally, the inhibition of ATP-dependent potassium channels enhanced the insulin secretion of hESC-derived ECCs (Fig. 4d), indicating that hESC-derived ECCs secret insulin via depolarization of K_ATP_ channels. Also, hESC-derived ECCs showed [Ca^2+^]*i* oscillation in the presence of high glucose levels (Fig. 4e and Supplementary Movie 2). Intriguingly, the first influx of [Ca^2+^] was detected immediately after glucose stimulation. This result suggests that hESC-derived ß cells in ECCs rapidly respond to glucose stimuli. In addition, membrane-docked insulin granules were observed in the cytoplasm of hESC-derived ECCs (Fig. 4f). From these results, it is likely that the exocytosis of insulin vesicles from hESC-derived ECCs occurs via [Ca2+]*i* oscillation. Collectively, our findings indicate that hESC-derived ECCs have ß cell-like properties in that they rapidly respond to glucose stimuli, showing both [Ca2+]*i* oscillation and insulin secretion.

This study presents the possibility that hPSC-derived ECCs can be potentially used for anti-diabetes treatment. The *in vivo* functionality of hPSC-derived ECCs has been tested in diabetic mouse models^14^,^17^,^18^,^19^,^43^. According to these reports, the therapeutic effects of transplanted hPSC-derived endocrine cells are inefficient, as it took a long period of time (over 40 days) to achieve the ability to secret insulin in response to glucose in diabetic mice. The requirement of a long period of time to achieve therapeutic effect of hPSC-derived endocrine cells might be attributed to the immaturity of hPSC-derived endocrine cells. In the present study, hESC-derived ECCs could secret insulin in response to glucose *in vitro* and normal glycemic control could be achieved within a short time (3 d) after transplantation of hPSC-derived ECCs in STZ-treated diabetic mice (Fig. 5a). These results indicate that hPSC-deived ECCs are functionally mature and additional maturation period for efficient insulin secretion *in vivo* is not required. Furthermore human pancreatic endocrine hormones were observed in the ECC-transplanted tissues (Fig. 5b), and human c-peptide was detected in the blood of ECC-transplanted mice (Fig. 5d). These results demonstrate that hESC-derived ECCs can quickly and effectively regulate blood glucose levels in diabetic mice. Thus, ECCs would be more effective for the β cell replacement therapy than ECs previously reported.

Unfortunately, transplanted hESC-derived ECCs were effective for the regulation of blood glucose for only a short time (approximately 12 d) post-operation. Blood glucose levels gradually increased from d 12 following the operation in most diabetic mice transplanted with hESC-derived ECCs (Fig. 5a). Out of 11 diabetic mice transplanted with hESC-derived ECCs, 2 died within 3 d, 4 survived for 4–12 d, and 5 survived more than 30 d post-operation (Supplementary Table 1). Incremental changes in blood glucose levels might be responsible for the decreased PDX1 activity or the reduced insulin production in the transplanted tissues. PDX1, a key ß cell maker, is essential for maintaining ß cell identity and functionality^44^, but it is translocated into the cytoplasm when inactivated^45^. PDX1 expression was detected in the cytoplasm of ECC-transplanted tissue autopsied 13 d after transplantation (Supplementary Figure 4b). Additionally, no PDX1 expression was observed in the cytoplasm of ECC-transplanted cells collected on d 49 after transplantation (Supplementary Figure 4c). Thus, it is conceivable that PDX1-expressing cells may affect the long-term survival of diabetic mice transplanted with hESC-derived ECCs. Appropriate microenvironments, including a hospitable extracellular matrix, oxygen gradients, nutrients, metabolites, and revascularization are required for successful islet transplantation treatments^46^,^47^. A variety of approaches, such as the use of bio-compatible scaffolds and improvement of the medium, should be tried in the near future to achieve long-term transplanted ECC functionality.

## Methods

### Culture of human pluripotent stem cells

The usage of human materials, including hPSCs, was approved by the KAIST Institutional Review Board (approval number KH2011-12). All methods were performed in accordance with the approved guidelines and informed consent was obtained from all subjects. Two human embryonic stem cell lines, i.e., WA01 (H1) hESCs^48^ and CHA15-hESCs^49^, as well as one hiPSC line^50^, were used in this study. The differentiation and analysis of other cell lines are presented in Supplementary Figure 5 for CHA15-hESC and Supplementary Figure 6 for hiPSCs. Human pluripotent stem cells (hPSCs) were maintained on mitomycin-C (MMC, A. G. Scientific, San Diego, CA, USA)-treated mouse embryonic fibroblast (MEF) feeder layers in ES medium at 37 °C with 5% CO_2_. The human PSC medium consisted of 80% basal DMEM/F12 (Invitrogen, Carlsbad, CA, USA) supplemented with 1.2 g/L sodium bicarbonate (Sigma, St. Louis, MO, USA), 1 mM L-glutamine (Sigma), 1% non-essential amino acids (Invitrogen), 1% penicillin-streptomycin (Invitrogen), 0.1 mM β-mercaptoethanol (Sigma), 20% Knock-Out™ serum replacement (KO-SR, Invitrogen), and 4 ng/ml FGF2 (R&D Systems, Minneapolis, MN, USA). The medium was changed daily. Undifferentiated hPSC colonies were passaged at a ratio of 1:3–1:4 through mechanical cutting into clumps using 10 mg/ml collagenase type IV (Invitrogen).

### Differentiation of hPSCs into pancreatic endocrine cells

Before the induction of differentiation, hPSC colonies were dissociated into single cells by being incubated in EDTA/PBS solution for 8 min at 37 °C, as previously described^51^. Dissociated cells were seeded at a density of 4 × 10^4^ cells/well on Matrigel-coated 4-well plates and then cultured in mTeSR medium (Stemcell Technologies, Vancouver, BC, Canada) supplemented with 10 μM Y27632 (ROCK inhibitor, A. G. Scientific) in a feeder-free system for 2 d. The overall protocol for the differentiation of hPSCs into endocrine cells is shown in Fig. 1a. For definitive endoderm (DE) induction, hPSCs were incubated in basal DMEM/F12 supplemented with 0.2% BSA (Sigma), 50 ng/ml activin A (R&D Systems), 3 μM CHIR99021 (Cayman Chemical, Ann Arbor, Michigan, USA) and 2 mM LiCl (Sigma) for 1 d. Then, the cells were cultured in basal DMEM/F12 supplemented with 0.2% BSA (Sigma), 1% B27 supplement (Invitrogen), and 50 ng/ml activin A (R&D Systems) for 4 d. Next, DE cells were cultured in Step II medium for 6 d to differentiate into pancreatic endoderm (PE) cells. Step II medium consists of DMEM (Invitrogen) supplemented with 0.5% B27 supplement (Invitrogen), 2 μM retionic acid (RA, Sigma), 2 μM dorsomorphin (A. G. Scientific), 10 μM SB431542 (Abcam Biochemicals, Cambridge, England), 5 ng/ml bFGF (Basic fibroblast growth factor, R&D Systems), and 250 nm KAAD-cyclopamine (Toronto Research Chemicals, Toronto, Canada). Then, PE cells were incubated in Step III medium for 4 d to differentiate into endocrine progenitor (EP) cells. Step III medium consists of DMEM containing 0.5% B27 supplement (Invitrogen), 50 μg/ml ascorbic acid (Sigma), 2 μM dorsomorphin (A. G. Scientific), 10 μM SB431542 (Abcam Biochemicals), and 10 μM DAPT (Abcam Biochemicals). The next step was to differentiate EP cells into endocrine cells (ECs). EP cells were cultured in Step IV medium for 8 d. Step IV medium consists of CMRL 1066 (Invitrogen) supplemented with 0.5% B27 supplement (Invitrogen), 0.5% penicillin–streptomycin (Invitrogen), 25 mM glucose (Sigma), 500 μM Dibutyryl-cAMP (Santa Cruz Biotechnology, Santa Cruz, CA, USA), 10 μM exendin-4 (Sigma), 2 μM dorsomorphin (A. G. Scientific), 10 μM SB431542 (Abcam Biochemicals), 10 mM nicotinamide (Sigma), and 50 μg/ml ascorbic acid (Sigma).

### Generation of endocrine cell clusters

For cluster formation, ECs were dissociated into single cells by treatment with Accutase (eBioscience, San Diego, CA, USA) at 37 °C for 15 min. To increase cell viability, the cells were treated with 10 μM Y27632 (A. G. Scientific). Dissociated ECs (5 × 10^4^ cells/well) were placed in each well of a non-coated 96-well plate (SPL Life Sciences, Pocheon, Korea) and incubated in Step IV medium for 1 d at 37 °C with 5% CO_2_.

### Quantitative RT-PCR

Total mRNA was extracted from hPSCs and differentiated cells using Easy-BLUE (Intron Biotechnology, Seongnam, Korea). Approximately 1 μg of total RNA was reverse-transcribed using M-MLV Reverse Transcriptase (Enzynomics, Daejeon, Korea) according to the manufacturer’s protocol. The expression level of each gene was measured using a 2 × Real-Time PCR kit (BioAssay, Deajeon, Korea) and analyzed using a CFX Connect Real-Time PCR Detection System (Bio-Rad Laboratories, Hercules, CA). The primers used in this study are listed in Supplementary Table 2. The reaction parameters for real-time RT-PCR analysis were as follows: 95 °C for 10 min, followed by 40 cycles of 95 °C for 30 sec, 50 or 60 °C for 30 sec, and 72 °C for 30 sec, with a final elongation step at 72 °C for 5 min. For relative expression analysis, the ΔCt value was calculated as the difference between the *GAPDH* Ct and the target Ct. Fold changes in gene expression levels between ECs and ECCs were determined using the formula 2^−(SΔCt−CΔCt)^.

### Flow cytometry

Differentiated cells were incubated with Accutase (eBioscience) at 37 °C for 10 min to dissociate them. After the cells were centrifuged at 300 × *g* for 5 min, the pellet was resuspended in FACS buffer (PBS containing 1% FBS). The dissociated cells were incubated with anti-CXCR4 (BD Biosciences, Bedford, MA, USA) and anti-PDX1 (Abcam) at 4 °C for 30 min, followed by an incubation with Alexa Fluor 488-conjugated donkey secondary antibodies (Invitrogen) at 4 °C for 15 min. Then, the cells were washed 5 times with FACS buffer. The samples were analyzed using a FACSCalibur flow cytometer (BD Biosciences). Data were evaluated using FlowJo software (Tree Star, Ashland, OR, USA).

### Immunohistochemistry

Cells were fixed in 4% formaldehyde (Sigma) for 30 min at room temperature (RT) and were then washed 3 times in PBS (Invitrogen) for 10 min. After the cells were fixed, they were permeabilized in 0.5% Triton X-100 (Sigma) for 30 min and then washed 3 times in PBS (Invitrogen) for 10 min. Next, the cells were blocked for 1 h in blocking solution containing 1% BSA (Sigma) or 3% normal donkey serum (Jackson ImmunoResearch, West Grove, USA) at RT and were then incubated with the indicated primary antibodies at 4 °C overnight. The primary antibodies used in this study are listed in Supplementary Table 2. After the samples were washed 5 times with PBS, they were incubated with Alexa Fluor 488- or 594-conjugated donkey secondary antibodies (diluted at a ratio of 1:500 in blocking solution) at RT for 1 h. DAPI (Invitrogen) was used to counterstain the nuclei.

For immunohistochemical staining of engrafted ECCs, the extracted tissues were fixed in 4% paraformaldehyde at RT for 4–6 h and then washed in distilled water for 2 h. The samples were embedded in paraffin using a Leica TP 1020 tissue processor (Leica, Germ Leica, Austria) and sectioned at a thickness of 5 μm using a Leica RM 2245 microtome (Leica). After the sections were deparaffinized and rehydrated, heat-induced epitope retrieval was performed in 10 mM citrate buffer with 0.05% Tween-20 (pH 6) for 10 min at 95 °C. The sections were immunostained as described above.

### Insulin secretion assay

Both ECs and ECCs were preincubated in Krebs-Ringer bicarbonate with HEPES buffer (KRBH; 115 mM NaCl, 24 mM NaHCO_3,_ 5 mM KCl, 2.5 mM CaCl_2,_ 1 mM MgCl_2_, 25 mM HEPES) supplemented with 2% BSA (Sigma) containing 2.5 mM glucose (Invitrogen) at 37 °C for 2 h for equilibration. After an additional incubation in fresh buffer for 1 h, cells were incubated in KRBH containing 27.5 mM glucose (Invitrogen) or 2.5 mM glucose (Invitrogen) with 30 mM KCl (Sigma), 100 μM tolbutamide (Sigma) at 37 °C for 1 h. Secreted insulin was measured from the supernatant. To measure total insulin, the cells were lysed in 0.5 ml of acid-ethanol by sonication on a Vibra-Cell sonicator™ (Sonics & Materials, Inc., Newtown, CT, USA) for 40 sec; the cells were then neutralized with the same volume of 1 M Tris-HCl (pH 7.5). Secreted insulin and total insulin were measured using an Ultrasensitive Insulin ELISA Kit (Alpco, Salem, USA) according to the manufacturer’s instructions. A Mercodia C-peptide ELISA Kit (Mercodia, Sylveniusgatan 8A, Sweden) was used to measure secreted human c-peptide after the same procedure described above.

### Ca^2+^imaging

ECCs were first attached on Matrigel (BD Biosciences)-coated cover-slips for 24 h. Attached ECCs were washed with PBS and then incubated in normal Tyrode’s solution (137 mM NaCl, 5.6 mM KCl, 10 mM HEPES, and 0.5 mM MgCl_2_) containing 2.5 mM glucose at 37 °C for 30 min. Then, 2 μM Fluo-4 AM (Invitrogen) and 0.008% Pluronic F-127 (Invitrogen) were loaded in fresh normal Tyrode’s solution for 20 min. Subsequently, ECCs were visualized using a fixed-stage microscope (Eclipse FN1, Nikon, Japan) for serial time-lapse imaging. Time-lapse imaging was acquired at 60 × magnification at intervals of 10 sec for 20 min. Glucose stimulation during imaging progressed as follows: 2 min in 2.5 mM glucose, 15 min in 27.5 glucose, and then 3 min in 2.5 mM glucose. The fluorescence intensity of the ECCs was analyzed using ImageJ software (NIH, imagej.nih.gov/ij/). The relative intensity of each spot was calculated by normalizing the fluorescence intensity from the average fluorescence intensity of individual cells within the cluster.

### Electron microscopy

ECCs were fixed in 3% glutaraldehyde in 0.1 M cacodylate buffer (pH 7.2) containing 0.1% CaCl_2_ for 3 h at RT and washed 5 times with 0.1 M cacodylate buffer at 4 °C. Then, the ECCs were postfixed in 1% OsO_4_ in 0.1 M cacodylate buffer containing 0.1% CaCl_2_ for 2 h at 4 °C. After the cells were washed with cold distilled water, they were dehydrated slowly with an ethanol series and propylene oxide at 4 °C. The samples were embedded in Embed-812 resin (EMS, Hatfield, PA, USA). After polymerization of the resin at 60 °C for 36 h, serial sections were cut with a diamond knife on an ULTRACUT UC7 ultramicrotome (Leica, Austria) and mounted on formvar-coated slot grids. The sections were stained with 4% uranyl acetate for 10 min and lead citrate for 7 min and were then observed using a Tecnai G2 Spirit Twin transmission electron microscope (FEI Company, Hillsboro, OR, USA).

### Real-time imaging

Live imaging of clustering was performed using a Chamlide WP system (Live Cell Instrument, Seoul, Korea) under humidified conditions at 37 °C with 5% CO_2_. Images of the clustering of dissociated ECs were captured at intervals of 5 min over 24 h using an Axio Vert.A1 microscope (Carl Zeiss).

### Transplantation of hESC-derived ECCs into immune-deficient mice

The performed animal care and experimental procedures were approved by the KAIST Animal Care Committee (approval number KA2013-29) and all methods were carried out in accordance with the approved guidelines.

For the transplantation experiment, male 4- to 10-week-old NOD.CB17-Prkdc^scid^/J mice were used. To generate the diabetic model, hyperglycemia was induced at 3 d before transplantation by a single injection of 175 mg/kg streptozotocin (STZ, Sigma). Lantus (Sanofi Aventis, Paris, France) was administered at dose of 0.15 U/kg for 2 d before transplantation. Mice were anesthetized by an intraperitoneal injection of 12.5 mg of Zoletil 50 (Virbac, S.A., France) and 3.886 mg of Rumpun (BAYER, Leverkusen, Germany)/kg of body weight. Approximately 2.2–3.8 × 10^4 ^ECCs mixed with Matrigel (BD Biosciences) were transplanted into the epididymal fat pad. To enhance hESC-derived ECC engraftment into the transplantation site, fibrous polycaprolactone (PCL) sheets were used. After transplantation, tail tip blood was collected every 3 d following 4 h of fasting; then, the glucose level was measured using a portable glucometer (Allmedicus Inc., Korea). Normal mice and STZ-treated, sham-operated mice (STZ + sham) were used as controls. STZ + sham mice were individually transplanted with 100 μL of a Matrigel and Step IV medium mixture into the epididymal fat pad. For serum preparation, mouse blood was taken from eye veins using capillary tubes (Fisher Scientific, MA, USA). After the blood samples were allowed to stand for 5 min at RT, the supernatant was collected to measure the level of human c-peptide.

### Statistical analysis

All statistical analyses were performed using Prism 5.01 (GraphPad Software, Inc., La Jolla, CA, USA). All data are shown as the mean ± SEM. The statistical significance of experimental outcomes was calculated using Student’s *t*-test. The differences between experimental groups were considered significant when *P* < 0.05.

## Additional Information

**How to cite this article**: Kim, Y. *et al.* Islet-like organoids derived from human pluripotent stem cells efficiently function in the glucose responsiveness *in vitro* and *in vivo. Sci. Rep.* **6**, 35145; doi: 10.1038/srep35145 (2016).

## Supplementary Material

Supplementary Information

Supplementary Movie 1

Supplementary Movie 2
