# Supplementary material for: Islet-like organoids derived from human pluripotent stem cells efficiently function in the glucose responsiveness in vitro and in vivo
Source: Sci Rep. 2016 Oct 12;6:35145. doi: 10.1038/srep35145 (PMC5059670; doi:10.1038/srep35145)
Supplement: Supplementary Information [file srep35145-s1.pdf]

Supplementary information for:

Islet-like organoids derived from human pluripotent stem cells efficiently function in the glucose responsiveness *in vitro* and *in vivo*

Youngjin Kim[a], Hyeongseok Kim[b], Ung Huyn Ko[c], Youjin Oh[a], Ajin Lim[a], Jong-Woo Sohn[a], Jennifer H. Shin[c], Hail Kim[b, *], and Yong-Mahn Han[a, *]

[a]Department of Biological Sciences; [b]Graduate School of Medical Science and Engineering; [c]Department of Mechanical Engineering, KAIST, Daejeon 314141, Republic of Korea.

Summary of Supplementary Information

Supplementary Figure 1: Immunostaining of PDX and pancreatic endocrine hormones in hESC-derived ECs

Supplementary Figure 2: Cell density of hESC-derived ECs for optimal clustering

Supplementary Figure 3: Expression of pancreatic endocrine hormones in hESC-derived ECCs and co-expression of GLUT1/PDX1 in orthogonal sections from hESC-derived ECCs

Supplementary Figure 4: Data obtained from diabetic mice transplanted with hESC-derived ECCs

Supplementary Figure 5: Generation of ECCs from another hESC line CHA15-hESC

Supplementary Figure 6: Generation of ECCs from human induced pluripotent stem cells (hiPSCs)

Supplementary Table 1: Survival of STZ-treated mice transplanted with hESC-derived ECCs

Supplementary Table 2: RT-PCR primer sequences

Supplementary Table 3: Primary antibody information

Supplementary Movie 1: Clustering of hESC-derived ECs

Supplementary Movie 2: Calcium oscillation in hESC-derived ECCs

Supplementary Methods

Supplementary Figure 1

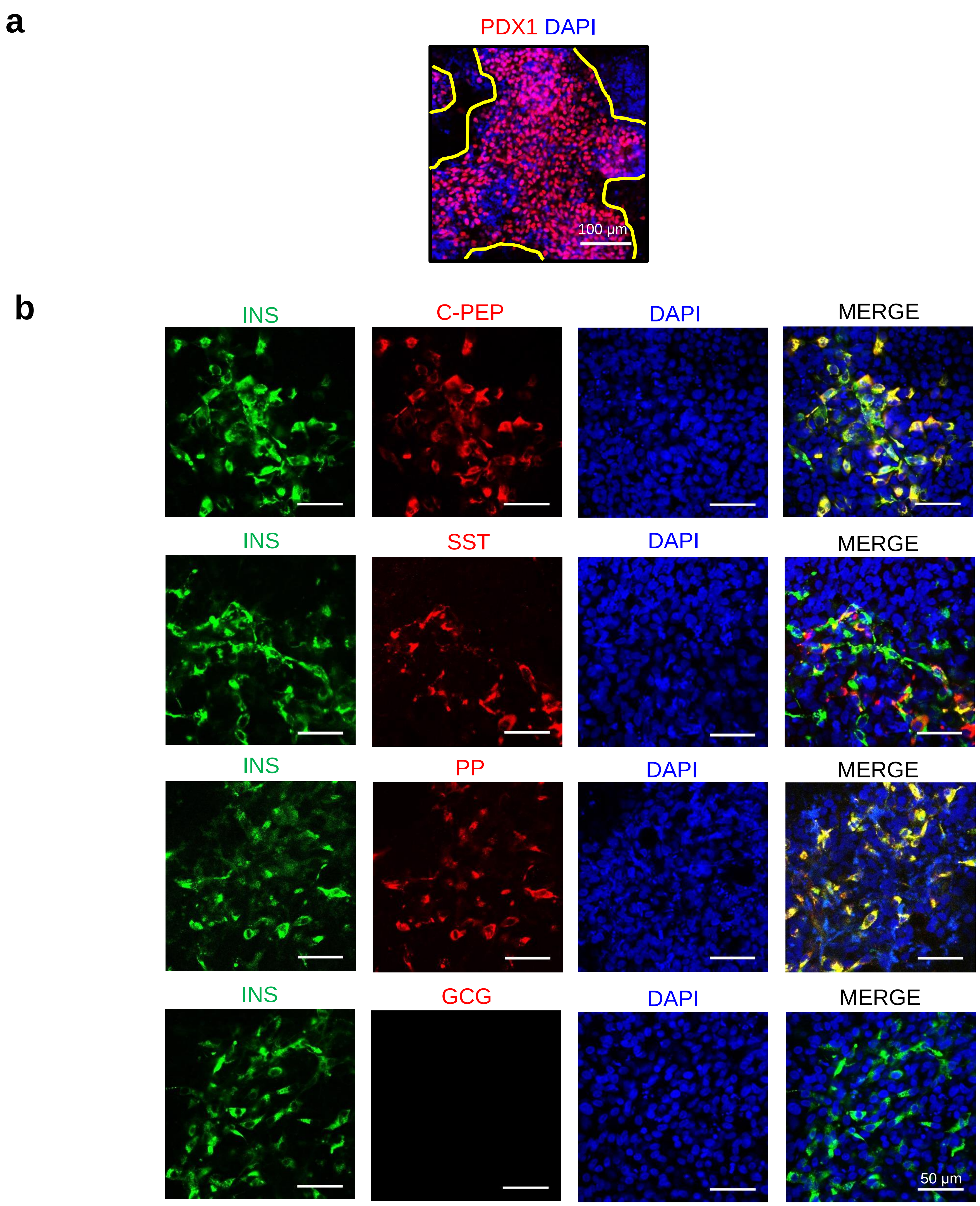

Supplementary Figure 1. Immunostaining of PDX and pancreatic endocrine hormones in hESC-derived ECs. (a) PDX1-expressing ECs (red) were randomly populated, as marked by a yellow line. Nuclear DAPI staining is shown in blue. Scale bar, 100 µm. (b) C-peptide (C-PEP) and pancreatic polypeptide (PP) were co-expressed in the insulin (INS)-expressing cells whereas glucagon (GCG) was not expressed in the insulin-expressing cells. And some of insulin-expressing cells expressed somatostatin (SST). Scale bar, 50 µm.

Supplementary Figure 2

Cell density (Cells / well)				
$5x10^3$	$1x10^4$	$5x10^4$	$1x10^5$	$5x10^5$
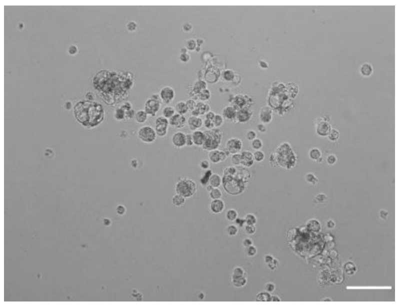	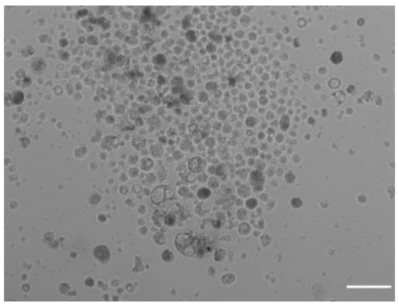	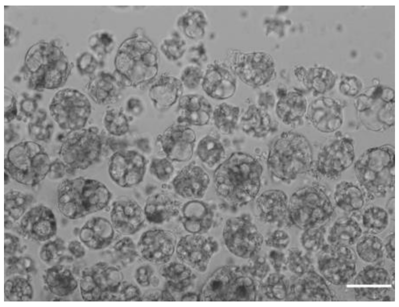	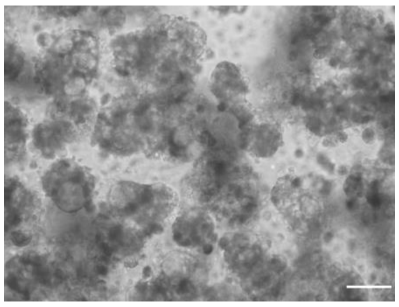	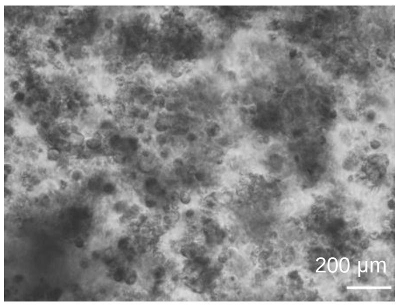

Supplementary Figure 2. Cell density of hESC-derived ECs for optimal clustering. Representative images of clusters generated from dissociated hESC-derived ECs were captured 24 h after seeding. Scale bar, 200 µm.

Supplementary Figure 3

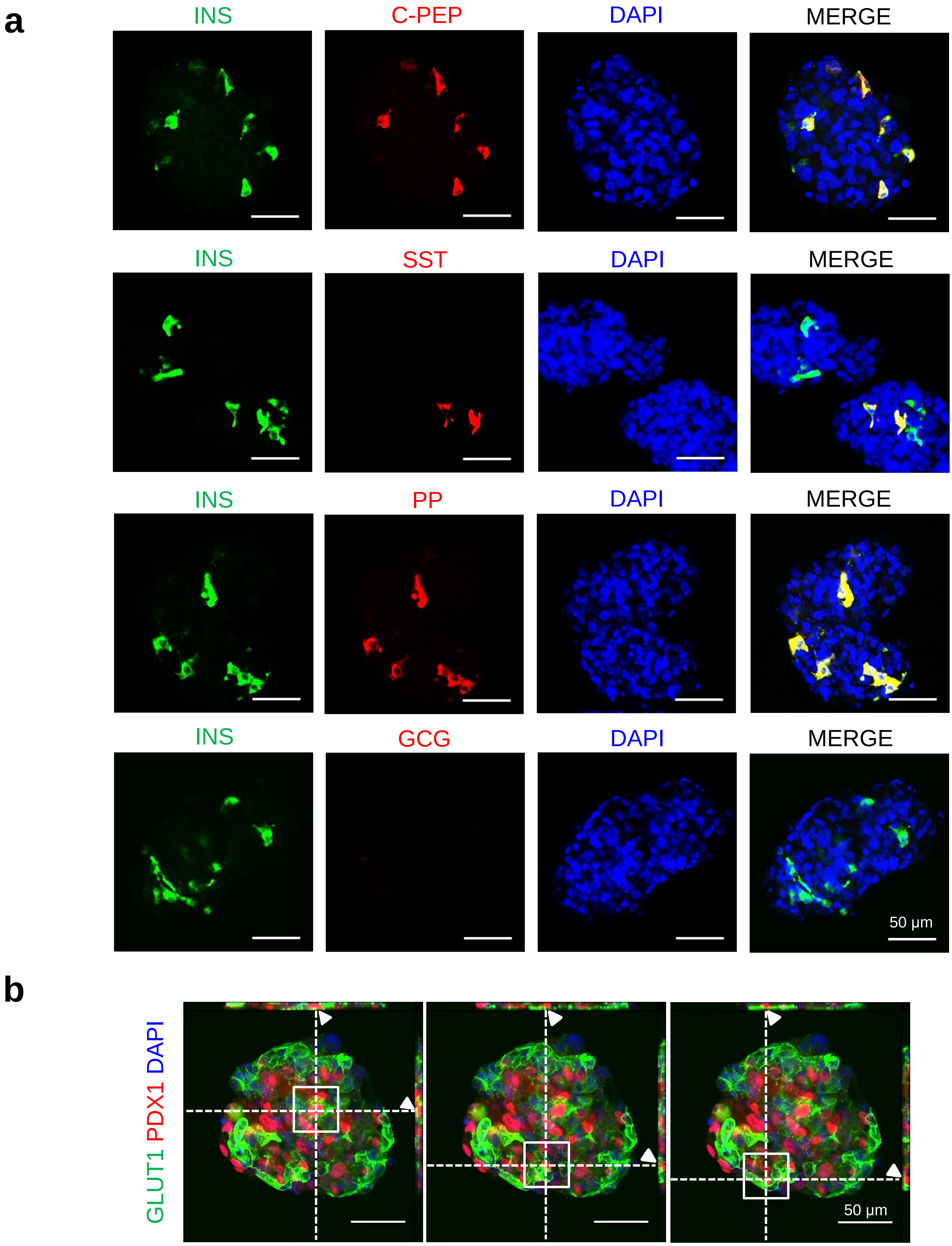

Supplementary Figure 3. Expression of pancreatic endocrine hormones in hESC-derived ECCs and co-expression of GLUT1/PDX1 in orthogonal sections from hESC-derived ECCs. (a) C-peptide (C-PEP) and pancreatic polypeptide (PP) were co-expressed with insulin (INS) in hESC-derived ECCs. And somatostatin (SST) was co-expressed in some of insulin-expressing cells. However, glucagon (GCG) was not expressed in the hESC-derived ECCs. Nuclear DAPI staining is shown in blue. Scale bar, 50 μm. (b) Orthogonal images were captured using a confocal z-stack system. Scale bar, 50 μm.

Supplementary Figure 4

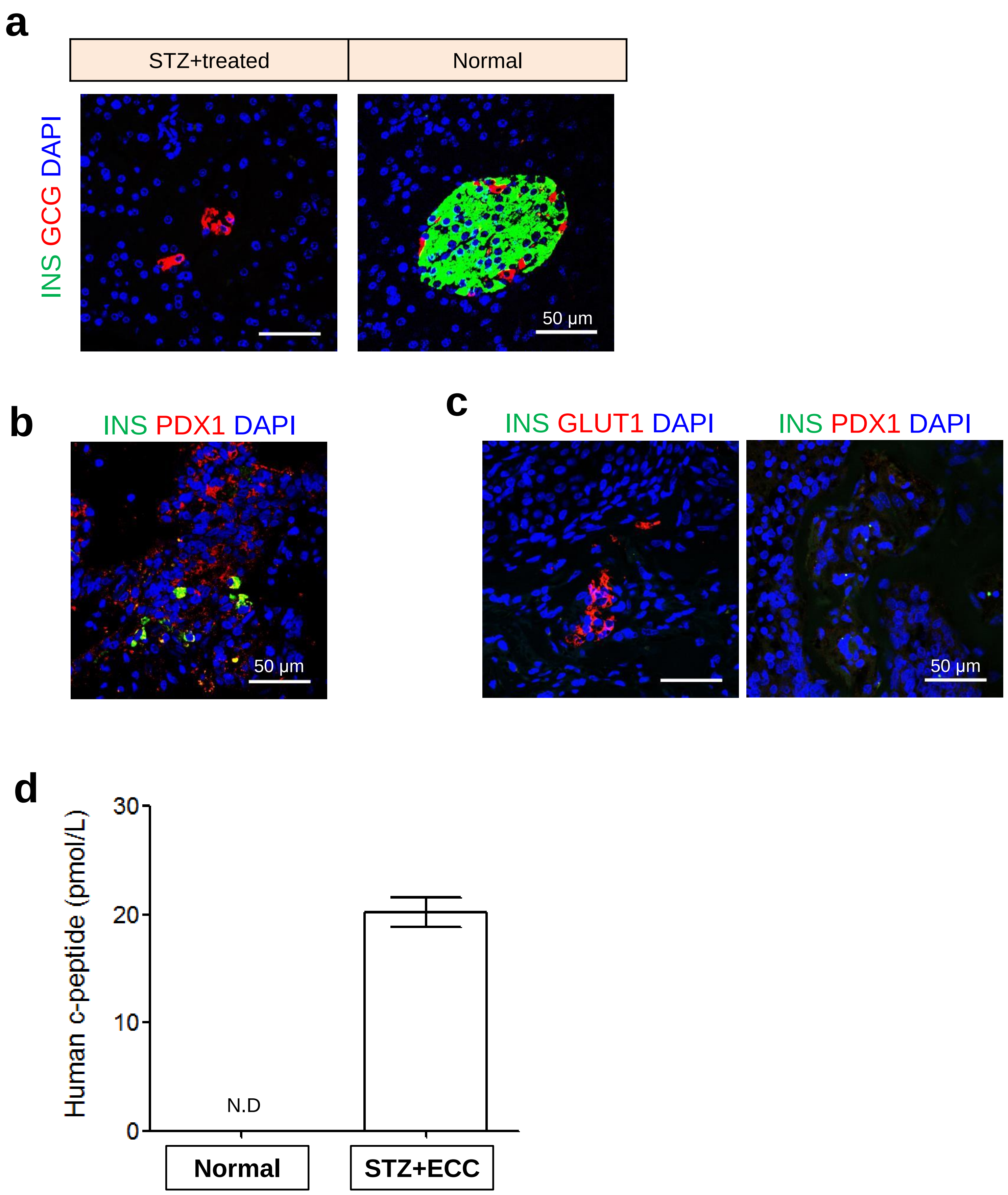

Supplementary Figure 4. Data obtained from diabetic mice transplanted with hESC-derived ECCs. (a) Pancreatic tissues of immune-deficient mice treated with streptozotocin (STZ). STZ-treated mice had completely destroyed pancreatic islets (right) compared with those of non-treated mice (left). The pancreatic islets are indicated by the co-immunostaining of INS/GCG. Nuclear DAPI staining is shown in blue. Scale bar, 50 μm. (b) Aberrant expression of PDX1 in ECC-transplanted tissue autopsied at 13 d post-operation. Scale bar, 50 μm. (c) No expression of INS and PDX1 was observed in ECC-transplanted tissue autopsied at 49 d post-operation. Scale bar, 50 μm (d) Human c-peptide secretion in the serum of mice transplanted with hESC-derived ECCs. The human c-peptide level was decreased in hyperglycemic mice at 15 d after the transplantation of hESC-derived ECCs. Measured c-peptide levels (pmol/L) are represented as the mean ± SEM (n=2). N.D., not detected.

Supplementary Figure 5

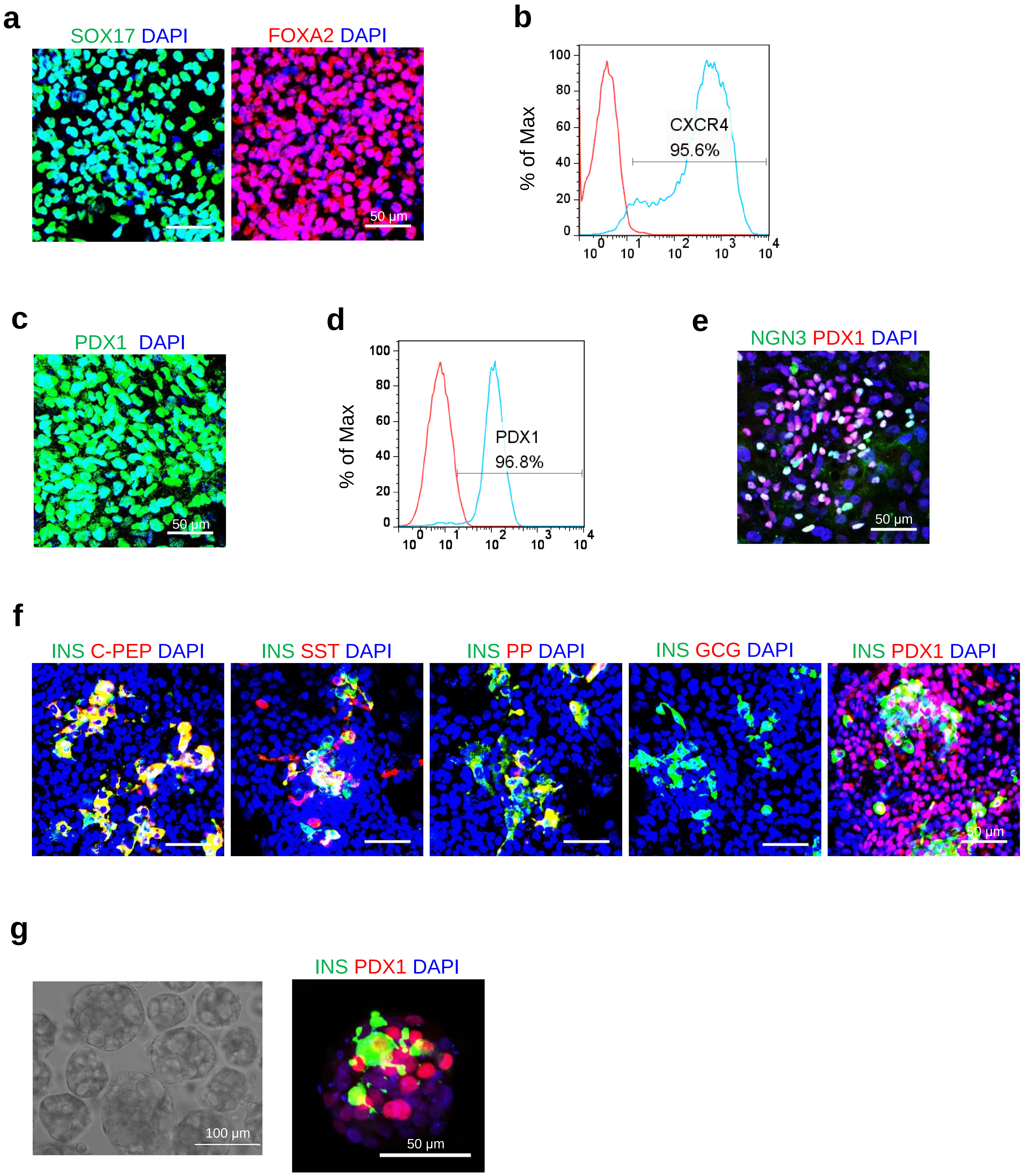

Supplementary Figure 5

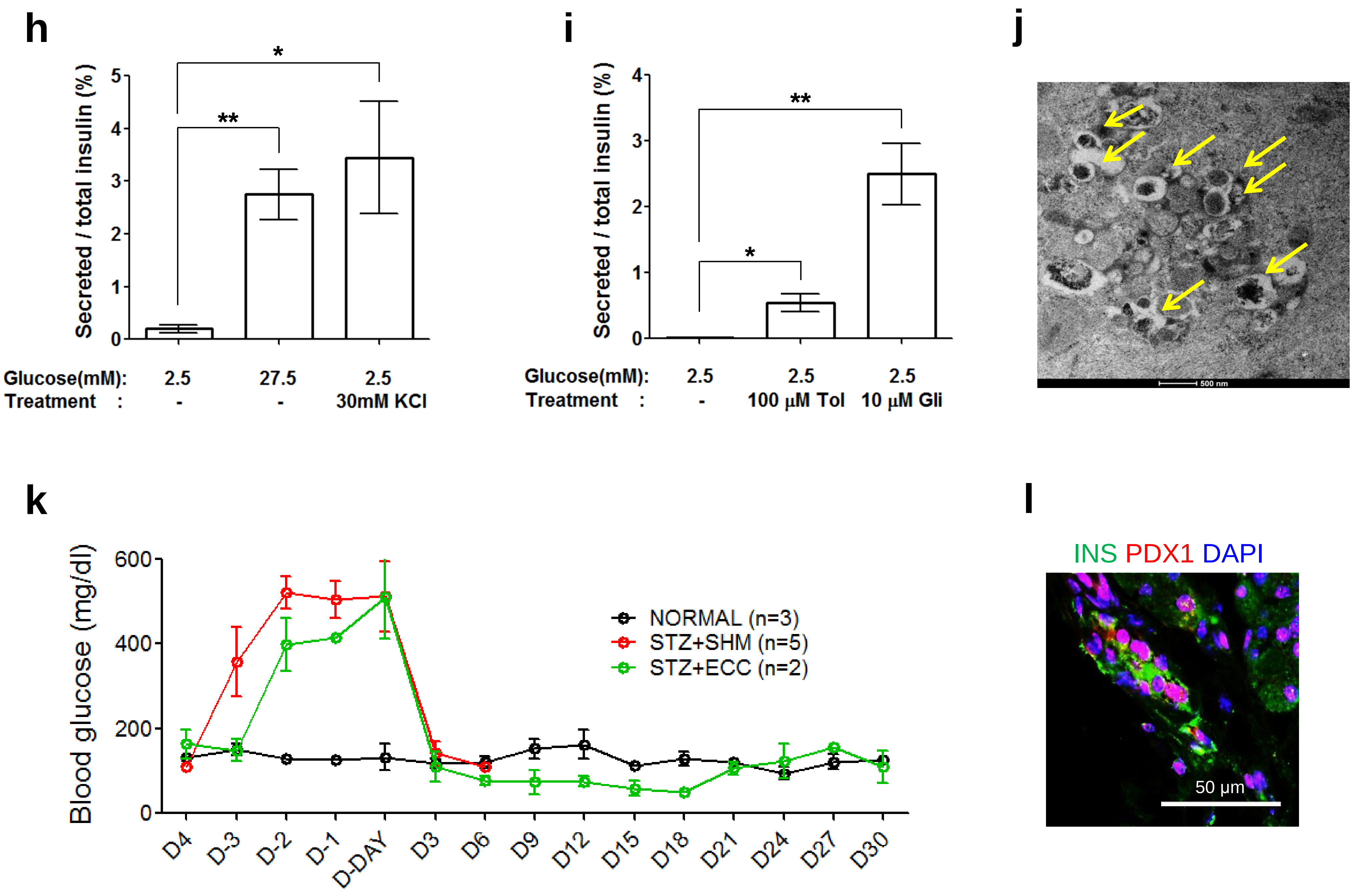

Supplementary Figure 5. Generation of ECCs from another hESC line, CHA15-hESC. (a) Differentiation of hESCs into DE cells. Immunostaining of the representative DE markers SOX17, and FOXA2 in hESC-derived DE cells. Nuclear DAPI staining is shown in blue. Scale bar, 50 µm. (b) Flow cytometric analysis of CXCR4-positive cells differentiated from hESCs. (c) Derivation of PE cells from hESC-derived DE cells. Immunostaining of PDX1 in hESC-derived PE cells. Scale bar, 50 µm. (d) Flow cytometric analysis of PDX1-positive cells in differentiated PE cells. (e) Co-immunostaining of hESC-derived EPs for NGN3/PDX1. Scale bar, 50 µm. (f) Expression of pancreatic endocrine hormones in CHA15-derived ECs. Scale bars, 50 µm. (g) Generation of ECCs from CHA15-derived ECs. Representative image of hESC-derived ECCs. CHA15-derived ECCs expressed INS/PDX1. (h-i) Enhanced insulin secretion from CHA15-derived ECCs in response to 27.5 mM glucose and 30 mM KCl (h), and 100 µM tolbutamide and 10 µM glimepiride (i). Secreted insulin levels are represented as the mean ± SEM (n=3 or 4); * $p < 0.05$, ** $p < 0.01$. (j) Transmission electron microscopy images of insulin-containing granules in CHA15-derived ECCs. Insulin-containing granules are indicated by yellow arrows. Scale bar, 500 nm. (k) Therapeutic effect of CHA15-derived ECCs in STZ-treated diabetic mice. Compared to STZ-treated controls (STZ+SHM, n=5), ECC-transplanted mice (STZ+ECC, n=2) were rescued from hyperglycemia right after transplantation and maintained normal glucose levels for 30 d. Blood glucose levels in the normal control group (normal, n=3) were also tracked during same period. The data are represented as the mean ± SEM. (l) Expression of INS/PDX1 in ECC-transplanted tissue. Scale bar, 50 µm.

Supplementary Figure 6

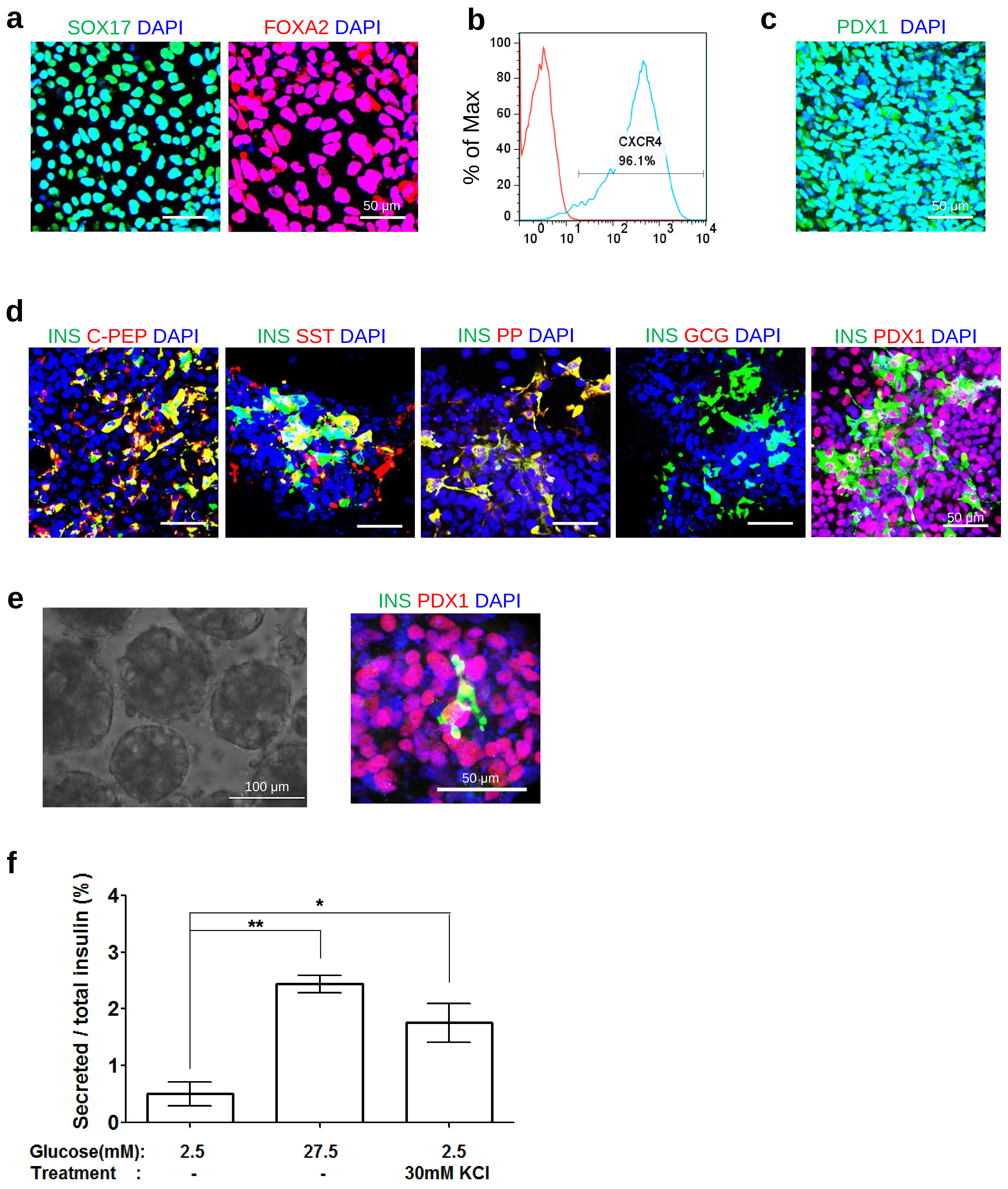

Supplementary Figure 6. Generation of ECCs from human induced pluripotent stem cells (hiPSCs). (a) Immunostaining of the representative DE markers SOX17, and FOXA2 in hiPSC-derived DE cells. Nuclear DAPI staining is shown in blue. Scale bar, 50 μm. (b) Flow cytometric analysis of CXCR4-positive cells differentiated from hiPSCs. CXCR4 was used as a DE marker. (c) Immunostaining of PDX1 in hiPSC-derived PE cells. Scale bar, 50 μm. (d) Expression of pancreatic endocrine hormones by hiPSC-derived ECs. Scale bars, 50 μm. (e) Representative image of hiPSC-derived ECCs. Scale bar, 100 μm (left). Co-expression of INS/PDX1 in hiPSC-derived ECCs. (f) Enhanced glucose responsiveness in hiPSC-derived ECCs. Secreted insulin levels are represented as the mean ± SEM (n=3); * $p < 0.05$, ** $p < 0.01$.

Supplementary Table 1. Survival of STZ-treated mice transplanted with hESC-derived ECCs

Group	Survival (d) post operation		
	0~3	4~12	>30
STZ+ECC (n)	2	4	5

hESC-derived ECCs were transplanted into the epididymal fat pads of diabetic mice. hESC-derived ECC-transplanted mice were divided into three groups, according to survival period (i.e., 0-3 d, 4-12 d, and more than 30 d).

Supplementary Table 2. RT-PCR primer sequences

Stage	Target	Forward sequence	Reverse sequence	Size
	GAPDH	CTTCGCTCTCTGCTCCTCCT	GTTAAAAGCAGCCCTGGTGA	152
Definitive endoderm	*SOX17*	CAGAATCCAGACCTGCACAA	GCGGCCGGTACTTGTAGTT	154
	GATA4	TCCAAACCAGAAAACGGAAG	CTGTGCCCGTAGTGAGATGA	187
	FOXA2	AACAAGATGCTGACGCTGAG	CAGGAAACAGTCGTTGAAGG	126
Pancreatic endoderm	*PDX1*	GTTCCGAGGTAGAGGCTGTG	AACATAACCCGAGCACAAGG	250
	SOX9	TACGACTACACCGACCACCA	TCAAGGTCGAGTGAGCTGTG	213
	HNF1β	AGCCCACCAACAAGAAGATG	CATTCTGCCCTGTTGCATTC	145
Endocrine lineage	*INS*	TGTACCAGCATCTGCTCCCTCTA	TGCTGGTTCAAGGGCTTTATTCCA	122
	SST	CCCCAG ACT CCG TCA GTTTC	TCC GTC TGG TTG GGT TCAG	108
	PPY	ACCTGCGTGGCTCTGTTACT	TACCTAGGCCTGGTCAGCAT	152
	GCG	AGGCAGACCCACTCAGTGA	AACAATGGCGACCTCTTCTG	308
	NGN3	AGAGCCTCGGAAGACGAAGT	AGTCAGCGCCCAGATGTAGT	342
	NKX2.2	TGGCCATGTAAACGTTCTGA	GGAAGAAAGCAGGGGAAAAC	189
	NKX6.1	ATTCGTTGGGGATGACAGAG	TGGGATCCAGAGGCTTATTG	186
	MAFB	CTCGTTTCTGATGCAGGACA	TGCAACTTTCAAGTGCGTTC	378
	MAFA	CTTCAGCAAGGAGGAGGTCATC	CTCGTATTTCTCCTTGTACAGGTCC	208
	PCSK1	CACATGGGGAGAGAACCCTA	ACGAGGCTGCTTCATATGCT	145
	SLC2A1	GCAACGGCTTAGACTTCGAC	TGCGACTTCAGGCACATAAC	283
	GCK	CTCAGTGAGGAAGGGTCCAG	CTGCCAGGATCTGCTCTACC	297
	CX36	TTCCTAGCCCTGGACAGAGA	AGCACCCCATTCACAATAGC	125
	CDH1	CTTGTTCTGAGTAAGTGTGTTC	TCATAGTTCCGCTCTGTCT	117

Supplementary Table 3. Primary antibody information

Stage	Target	Host	Dilution used	Company
Definitive endoderm	SOX17	Goat	1 : 400	R&D Systems, AF1924
	FOXA2	Rabbit	1 : 400	Cell Signaling, 3143S
	GATA4	Mouse	1 : 400	Santa Cruz, sc-25310
Pancreatic endoderm	PDX1	Rabbit	1 : 1000	Abcam, ab47267
Endocrine lineage	INSULIN	Guinea pig	1 : 500	Dako, A0564
	C-PEPTIDE	Mouse	1 : 1000	Abcam, ab8297
	SOMATOSTATIN	Rabbit	1 : 1000	Dako, A0566
	PANCREATIC POLYPEPTIDE	Goat	1 : 500	Sigma, SAB2500747
	GLUCAGON	Mouse	1 : 500	Sigma, G2654
	NGN3	Mouse	1 : 50	DSHB, F25A1B3
	NKX2.2	Mouse	1 : 10	DSHB, 74.5A5
	NKX6.1	Rabbit	1 : 50	Sigma, HPA036774
	MAFB	Rabbit	1 : 100	Bethyl, IHC-00351
	PC1/3	Mouse	1 : 200	Abcam, ab55543
	GLUT1	Rabbit	1 : 500	Thermo Scientific, RB-9052
	GLUT1	Mouse	1 : 500	Abcam, ab40084
	E-CAD	Mouse	1 : 500	BD Biosciences, 610181

Supplementary Movie 1. Clustering of hESC-derived ECs

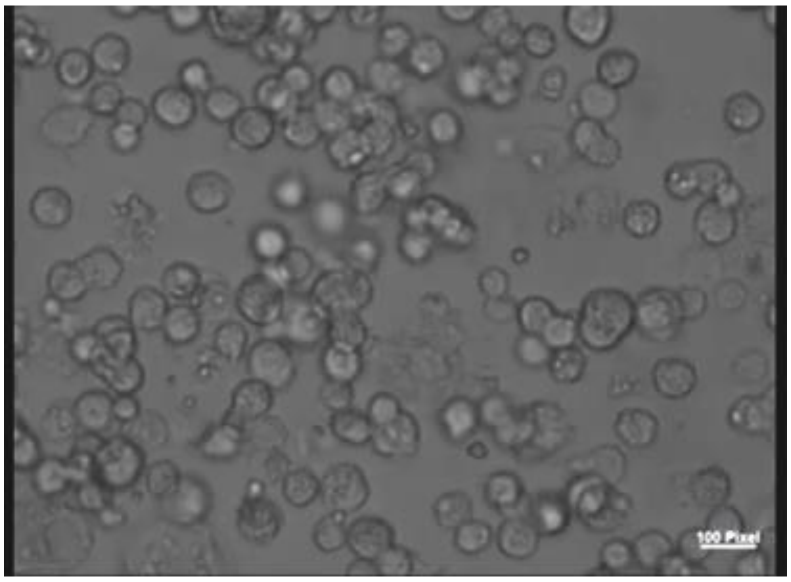

The clustering process of dissociated hESC-derived ECs was recorded for 24 h. Serial images were captured every 5 min. The humidified culture conditions were maintained throughout the recording period at 37°C with 5% CO_2. Scale bar, 100 pixels.

Supplementary Movie 2. Ca^{2+} influx in hESC-derived ECCs during glucose stimulation.

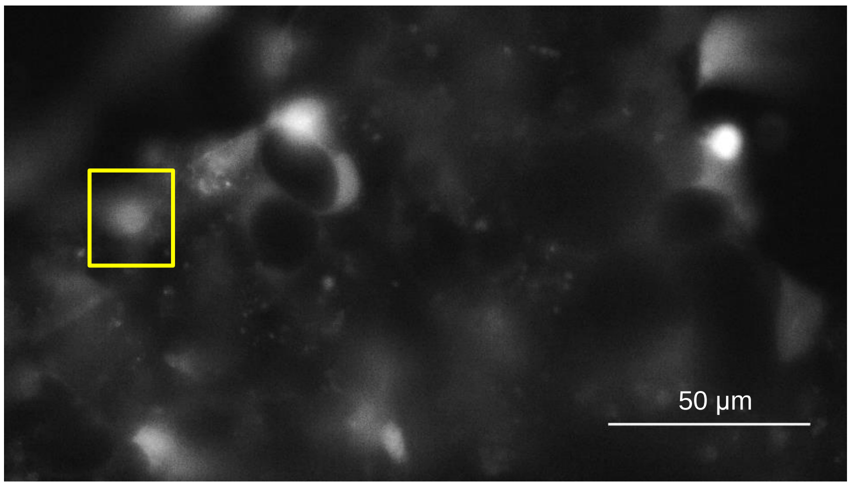

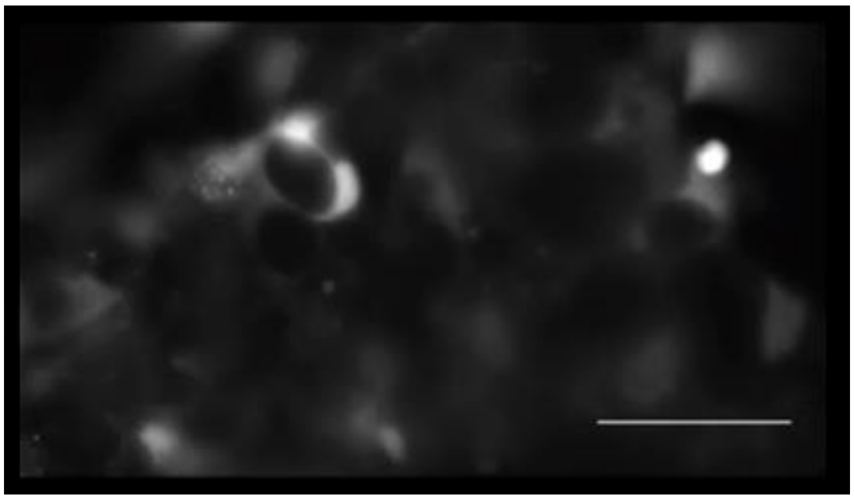

The cytosolic Ca^{2+} level was detected using Fluo-4 AM (Invitrogen). Serial images were captured every 10 sec for 20 min. After 2 min in 2.5 mM glucose, the ECCs were stimulated with 27.5 mM glucose for 15 min and then stabilized in 2.5 mM glucose for the remaining 3 min. The representative functional fraction of ECCs is indicated by the yellow box. Scale bar, 50 μm.

Supplementary Methods

Electrospinning

To obtain fibrous polycaprolactone (PCL, Sigma) sheets, PCL pallets (80,000 MW) were dissolved (20% (w/v) in a mixed solution of dichloromethane (DM, Junsei, Japan) and N,N-dimethylformamide (NDF, Junsei, Japan) at a 1:3 ratio. The PCL solution was infused from a 6-mL disposable syringe at a volume flow rate of 3 mL/h through a 25-G syringe needle at 13 kV to electrospin PCL fibers 1-3 μm in diameter. The syringe needle was positioned at 275 mm above the aluminum substrate, and the fibers were collected for 3 min. The input voltage and needle size together determined the fiber thickness, while the distance to the collector along with the flow rate influence the uniformity of the fibers by controlling the evaporation of the solvent during the fabrication process. The collection time was chosen to obtain the optimal density of fibers in the sheet, i.e., one that is desirable for physically constraining the spheroids, while ensuring sufficient transport of necessary molecules.

Transplantation of hESC-derived ECCs into the kidney capsule

Approximately 1.7 × 104 – 1.9 × 104 ECCs were transplanted into the kidney capsule using Intramedic™ polyethylene tubing (PE 50, BD Biosciences). After transplantation, tail tip blood was collected every 3 d following fasting for 4 h, and the glucose level was then measured using a portable glucometer (Allmedicus Inc., Korea).

Generation and maintenance of iPS cells

The performed protocols in this study were approved by the institutional review board of the Asan Medical Center, and written informed consent was obtained from the patient. To induce pluripotency, Yamanaka factors (*OCT4*, *SOX2*, *c-MYC* and *KLF4*) were introduced into fibroblasts by retroviral infection. At 5 days after infection, the fibroblasts were transferred onto a mitomycin C (MMC, A.G. Scientific)-treated MEF feeder layer and cultured for approximately 2-3 weeks, until colonies formed. Single colonies were selected and separately cultured as individual cell lines. iPSCs were cultured in human embryonic stem cell (hESC) medium at 37°C and 5% CO_2 in air. The hESC medium consisted of DMEM/F12 (Invitrogen) supplemented with 1.2 g/L sodium bicarbonate (Sigma), 1 mM L-glutamine (Sigma), 1% non-essential amino acid (Invitrogen), 1% penicillin/streptomycin (Invitrogen), 0.1 mM ß-mercaptoethanol (Sigma), 20% KO-SR, and 10 ng/mL bFGF (R&D Systems). Every 5 days, single iPSC colonies were mechanically sliced into 10-15 sections and detached after incubation with 0.1 mg/mL collagenase type IV (Invitrogen) for 4 min. The medium was changed daily.
